# Xenon LFP Analysis Platform Is a Novel Graphical User Interface for Analysis of Local Field Potential From Large-Scale MEA Recordings

**DOI:** 10.3389/fnins.2022.904931

**Published:** 2022-07-01

**Authors:** Arjun Mahadevan, Neela K. Codadu, R. Ryley Parrish

**Affiliations:** ^1^Department of Cellular and Molecular Biology, Xenon Pharmaceuticals Inc., Burnaby, BC, Canada; ^2^Department of Clinical and Experimental Epilepsy, Institute of Neurology, University College London, London, United Kingdom

**Keywords:** SCN1a, seizures, LFP analysis, Plotly Dash, HD-MEA

## Abstract

High-density multi-electrode array (HD-MEA) has enabled neuronal measurements at high spatial resolution to record local field potentials (LFP), extracellular action potentials, and network-wide extracellular recording on an extended spatial scale. While we have advanced recording systems with over 4,000 electrodes capable of recording data at over 20 kHz, it still presents computational challenges to handle, process, extract, and view information from these large recordings. We have created a computational method, and an open-source toolkit built in Python, rendered on a web browser using Plotly’s Dash for extracting and viewing the data and creating interactive visualization. In addition to extracting and viewing entire or small chunks of data sampled at lower or higher frequencies, respectively, it provides a framework to collect user inputs, analyze channel groups, generate raster plots, view quick summary measures for LFP activity, detect and isolate noise channels, and generate plots and visualization in both time and frequency domain. Incorporated into our Graphical User Interface (GUI), we also created a novel seizure detection method, which can be used to detect the onset of seizures in all or a selected group of channels and provide the following measures of seizures: distance, duration, and propagation across the region of interest. We demonstrate the utility of this toolkit, using datasets collected from an HD-MEA device comprising of 4,096 recording electrodes. For the current analysis, we demonstrate the toolkit and methods with a low sampling frequency dataset (300 Hz) and a group of approximately 400 channels. Using this toolkit, we present novel data demonstrating increased seizure propagation speed from brain slices of Scn1aHet mice compared to littermate controls. While there have been advances in HD-MEA recording systems with high spatial and temporal resolution, limited tools are available for researchers to view and process these big datasets. We now provide a user-friendly toolkit to analyze LFP activity obtained from large-scale MEA recordings with translatable applications to EEG recordings and demonstrate the utility of this new graphic user interface with novel biological findings.

## Introduction

The technology of neuronal data acquisition using high density multi-electrode arrays (HD-MEAs) in tissue and cell cultures has grown dramatically over the past decade (Maccione et al., 2013, 2014, 2015; Ingebrandt, 2015; Müller et al., 2015; Dragas et al., 2017; Viswam et al., 2017; Steinmetz et al., 2019; Paulk et al., 2022). A brief history of MEA technology and advancement of these devices is discussed extensively by Didier et al. (2020). These ever-growing, state-of-the-art electrophysiology techniques (Stevenson and Kording, 2011; Lopez et al., 2018; Miccoli et al., 2019) now include HD-MEA devices capable of recording extracellular neuronal signals from cell cultures or brain slices from thousands of electrodes (Ronchi et al., 2019). Pharmaceutical techniques have also expanded to include the high density multi-electrode arrays in new assay development (Kraushaar and Guenther, 2019). Several pharmaceutical applications and drug-testing protocols require long-duration recordings from 45 to 90 min (Codadu et al., 2019a), which can result in large data files of 350 to 500 GB.

While electrophysiology and chip technology progresses at a rapid pace generating high-quality precise neuronal data with a high degree of spatial accuracy, developing data analysis platforms and algorithms exploiting the full potential of the recordings is quite challenging (Mahmud and Vassanelli, 2016; Paninski and Cunningham, 2018). The progress in data analysis pipelines, big data algorithms, and flexible analysis platforms to adapt to different techniques, data formats, and research requirements is slowly evolving to handle the large scale of data (Landhuis, 2017). Most applications using high density MEA recordings rely on analysis of high-frequency activity, such as action-potential data, to include useful features, such as spike sorting, which has received a lot of attention in the research community, including several open-source architecture toolboxes to view and process the data (Pachitariu et al., 2016; Yger et al., 2018; Lee et al., 2020). Proprietary software and open-source toolboxes that come with the HD-MEA measurement systems can sometimes be restrictive to researchers. While they do provide blackbox-type solutions to spike identification, sorting, generating raster plots following spike sorting and other measures, they may not offer enough customization and adaptability to different methods of viewing and analyzing the data (Bridges et al., 2018). Moreover, while different toolboxes and software platforms provide different functionality, there are benefits and limitations related to the scalability of algorithms for large-scale data, and new paradigms are constantly evolving to exploit the vast potential of these recordings (Mahmud et al., 2012; Diggelmann et al., 2018; Sedaghat-Nejad et al., 2021; Buccino et al., 2022).

There are many options for analysis of extracellular action potentials for large-scale MEA recordings (Franke et al., 2015; Buccino et al., 2020; Petersen et al., 2021; Hu et al., 2022). However, open-source, user-friendly analysis platforms for visualizing long recordings of LFP collected from HD-MEA systems is limited. From our review of literature and open-source toolboxes, there are limited data-analysis pipelines that are flexible, customizable, and object-oriented methods for processing and visualizing data for low-frequency (0.5 to 300 Hz) LFP activity. This will continue to limit the usefulness of these large-scale MEA recording systems for many electrophysiologists. Nevertheless, there is an increasing number of research labs using HD-MEAs to record LFP activity to understand neuronal network dynamics from cortical brain slices (Ferrea et al., 2012; Toader et al., 2013; Medrihan et al., 2015; Hu et al., 2022). One available toolbox to view MEA data is presented by Bridges et al. (2018), built using Python leveraging GPU (Graphics Processing Units) capabilities to view and generate visualization for large MEA data files. However, this toolbox is only for viewing select traces from MEA recordings, is not maintained, and does not have any filtering or analysis features within the framework. In our current work, we present a much different data pipeline built in Python with diverse features and summary metrics rendered on a browser using Plotly’s Dash. This data-analysis pipeline is for band-pass filtered (0.5 to 2,048 Hz) LFP activity and seizure analysis that is scalable to large datasets, with an interactive GUI for analyzing HD-MEA measurements. This GUI includes several features to generate summary measures and plots, and trace LFP activity over time. For people familiar with basic Python, this tool can also serve as a framework to customize and add functions and visualization based on individual researchers’ analysis requirements.

Researchers also require novel ways to track LFP activity over space and time, as calcium imaging is limited by slow kinetics (Tang et al., 2015; Helassa et al., 2016; Vanwalleghem et al., 2020; Wei et al., 2020) and current voltage-imaging techniques have several weaknesses, such as high-bleaching properties (Kulkarni and Miller, 2017; Xiao et al., 2021). Recordings using high-resolution MEA systems offer a new way to explore network communication with a high degree of time and spatial resolution but require tools to tap into their full potential. Our new data pipeline offers an efficient and easy tool to analyze the spatial and time resolution offered by these MEA systems. We demonstrate the utility of this data pipeline with induction of seizure-like activity and generating example LFP raster plots over time and space, along with example traces from subregions of the brain. This bird’s-eye view of LFP activity within our GUI creates a new tool for investigation into novel insights into network dynamics, such as how the neocortex and hippocampus interact with each other. Furthermore, we demonstrate a novel seizure-tracking approach using the high density of electrophysiological channels with potential to be superior to large-scale calcium imaging to track seizure dynamics. We present data using this analysis tool that shows brain slices from Scn1aHet mice with a deficit in sodium channel NaV1.1, an important channel for interneuron excitability, have more seizure-like events (SLE) than wild-type (WT) littermates in a low Mg^2+^ model. Furthermore, we show novel data that demonstrate an increased seizure-propagation rate in the Scn1aHet mice, likely due to the well-documented decreased firing rates of parvalbumin-positive interneurons in these mouse models (Martin et al., 2010; Tai et al., 2014; Favero et al., 2018). We provide this new python-based software tool as an open-source, customizable solution for analysis and tracking of LFP activity using the 3Brain MEA recording system, but it can easily be adapted to any MEA recording platform. This GUI will also likely be suitable for analysis of large-scale EEG recordings and provide a useful mapping tool for *in vivo* LFP activity. Our current GUI has a particular utility for analysis of seizure-like activity but can be used for analysis of many other network LFP signals.

## Materials and Methods

### Ethical Approval

All animal handling and experimentation involving animals were conducted following approved protocols according to the guidelines of the Canadian Council on Animal Welfare and approved by the Xenon Animal Care Committee (XACC).

### Brain Slice Preparation

Heterozygous *scn1a* [Scn1a(+/-)] mice (Mistry et al., 2014) and WT littermates were used in this study. Heterozygous mice on the 129S6/SvEvTac background (MMRC strain number 037107) are crossed with C57BL/6 mice at The Jackson Laboratory (Ben Harbor, ME). The pregnant mice are then shipped to Xenon Pharmaceuticals to litter. Pups are then genotyped to determine their genotype as either WT for the *Scn1a* gene (Scn1a+/+) or heterozygous for the *Scn1a* gene (Scn1a+/-). All mice used in the study were genotyped a second time on the day of euthanasia to reconfirm their genotype. Scn1a and WT mice were used in this study between the ages of P21-P28. Mice were housed in individually ventilated cages in 12 h light, 12 h dark lighting regime. Animals received food and water *ad libitum*. Mice were anesthetized with isoflurane before being euthanatized by cervical dislocation. Brains were then removed and stored in cold cutting solution (in mM): 3 MgCl2; 126 NaCl; 26 NaHCO3; 3.5 KCl; 1.26 NaH2PO4; 10 glucose. For multi-electrode array recordings, 350 μm horizontal brain slices containing both the neocortex and hippocampus were made, using a Leica VT1200 vibratome (Nussloch, Germany). Brain slices were then transferred to a holding chamber and incubated for 1–2 h at room temperature in artificial CSF (ACSF) containing (in mM): 2 CaCl2; 1 MgCl2; 126 NaCl; 26 NaHCO3; 3.5 KCl; 1.26 NaH2PO4; 10 glucose. All the solutions were bubbled continuously to saturate with carboxygen (95% O2 and 5% CO2).

Multi-electrode array recordings were performed on the 3Brain BioCAM DupleX system (Switzerland) using the 3Brain Accura HD-MEA chips with 4,096 electrodes at a pitch of 60 μm. Brain slices were placed onto the electrodes with a harp placed on top to keep the slice pressed down gently to the recording electrodes. The slices were first incubated in the recording chamber for 10 min in ACSF. Following the 10-min incubation in ACSF, slices were then perfused in ACSF that had Mg^2+^ lowered to 25 μM to induce epileptiform-like activity. This allowed us to record the entire evolution of the induced epileptiform-like activity, ensuring we were able to record the first seizure-like events from every slice. Recordings were obtained from the entire slice, containing both the neocortex and the hippocampus. Experiments were performed at 33–36°C. The solutions were perfused at the rate of 5.0 mL/min. Signals were sampled at 10 kHz with a high-pass filter at 2 Hz.

### Statistics

Statistics were done in GraphPad Prism 9.1.1 (San Diego, CA, United States). Data was first checked for normal distribution using a Shapiro–Wilk normality test. Nonparametric data was analyzed with a Mann–Whitney test, and the parametric data was analyzed with an unpaired Student’s *t*-test. GraphPad Prism was used to graph scatter-point data. Significance was set at *P* ≤ 0.05 for all analyses.

### Data Analysis and Figures

The analysis platform and algorithms used were custom written in Python, including NumPy, pandas, SciPy, and visualizations using Plotly’s Dash libraries. The code and sample data files are provided through a GitHub repository^1^. Figures for the manuscript were created using diagrams, Inkscape 1.1.

### Performance

Xenon LFP Analysis Platform is a Python based Plotly Dash application rendering an optimized, interactive web-interface with relatively quick responsiveness. Benchmarking was performed to assess the overall performance of the Xenon LFP Analysis Platform (Table 1) on files of varied sizes, going up to 7.2 GB. The total recording time for each file was 50 min, sampled at 300 Hz, with 898,952 datapoints per channel. For best performance speed, we would recommend keeping file sizes to under 25% of the computers installed RAM. Performance was assessed on a Windows Server 2019 Standard machine with an Intel Xeon Gold 6126 CPU @ 2.60 GHz (2 processors) and 64GB of RAM.

**TABLE 1:** Benchmarking numbers for Xenon LFP analysis platform.

File size (GB)	Active channels	File read time (s)	Raster output times (s)	Time to plot 3 traces (s)
0.17	100	4.43	5.41	4.91
0.50	300	11.81	15.90	7.10
1.12	600	37.83	48.90	10.90
2.22	1,200	73.59	82.44	18.93
4.43	2,500	135.90	158.66	43.31
7.20	4,096	300.01	316.26	63.11

*The total recording time for each file was ∼50 min (2,997 s), with a downsampled frequency of 300 Hz, consisting of 898,952 datapoints per channel.*

*The original measurement file had a total recording time of ∼50 min (2,997 s) at a sampling frequency of 10 kHz.*

### Channel Group Function

To generate data on subsets of channels within the GUI, groups of channels can be selected within the GUI for analysis. The channel group functions are useful for comparing two or three different regions of the brain slice and for comparing LFP activity summary measures from select brain regions of interest.

### Local Field Potential Measures in Channel Groups

LFP peak count per second: To detect local field potential from voltage traces, the signal processing library from SciPy in Python is used, specifically the “scipy.signal.find_peak” function. This peak find function is described in detail in the following documentation: https://docs.scipy.org/doc/scipy/reference/generated/scipy.signal.find_peaks.html. The properties for the peak, including amplitude and width, are also extracted from the function. The inputs to this function include the “threshold” and “width,” which are received as inputs from the user in the GUI as Threshold (mV) and Time Duration (s) respectively. The “find_peak” function returns the time index of peaks, which are the local maxima or minima points in the signal, that exceed the minimum threshold value for the minimum specified duration for each individual channel; no maximum limits are set. The sum of the count of the peaks for each channel in the group is calculated as follows:


L⁢F⁢P⁢m⁢e⁢a⁢n⁢p⁢e⁢a⁢k⁢a⁢m⁢p⁢l⁢i⁢t⁢u⁢d⁢e⁢(m⁢V)

$$=\frac{\sum A\,v\,e\,r\,a\,g\,e\,L\,F\,P\,p\,e\,a\,k\,a\,m\,p\,l\,i\,t\,u\,d\,e\,f\,o\,r\,e\,a\,c\,h\,c\,h\,a\,n\,n\,e\,l\,i\,n\,t\,h\,e\,g\,r\,o\,u\,p}{T\,o\,t\,a\,l\,n\,u\,m\,b\,e\,r\,o\,f\,c\,h\,a\,n\,n\,e\,l\,s\,i\,n\,t\,h\,e\,g\,r\,o\,u\,p}$$

LFP mean peak amplitude (mV): For each channel, the peak locations are the local maxima or minima points in the signal, the amplitude at each peak is the difference between the voltage at the maxima or minimal point to the baseline voltage. The amplitude for all the peaks in the signal is calculated, which is used to calculate the average LFP peak amplitude for each individual channel. For a group of channels, the LFP mean peak amplitude is calculated as follows:


L⁢F⁢P⁢m⁢e⁢a⁢n⁢p⁢e⁢a⁢k⁢a⁢m⁢p⁢l⁢i⁢t⁢u⁢d⁢e⁢(m⁢V)

$$=\frac{\sum A\,v\,e\,r\,a\,g\,e\,L\,F\,P\,p\,e\,a\,k\,a\,m\,p\,l\,i\,t\,u\,d\,e\,f\,o\,r\,e\,a\,c\,h\,c\,h\,a\,n\,n\,e\,l\,i\,n\,t\,h\,e\,g\,r\,o\,u\,p}{T\,o\,t\,a\,l\,n\,u\,m\,b\,e\,r\,o\,f\,c\,h\,a\,n\,n\,e\,l\,s\,i\,n\,t\,h\,e\,g\,r\,o\,u\,p}$$

LFP mean peak duration (s): For each channel, the width of each peak is extracted using “properties” in the “find_peak” function. The duration for each peak location is the full width of the peak (in seconds), at the baseline of the signal, which is used to calculate the average LFP duration (width) for each individual channel in seconds. For a group of channels, the LFP peak mean duration is calculated as follows:


L⁢F⁢P⁢m⁢e⁢a⁢n⁢p⁢e⁢a⁢k⁢d⁢u⁢r⁢a⁢t⁢i⁢o⁢n⁢(s)

$$=\frac{\sum A\,v\,e\,r\,a\,g\,e\,L\,F\,P\,p\,e\,a\,k\,d\,u\,r\,a\,t\,i\,o\,n\,f\,o\,r\,e\,a\,c\,h\,c\,h\,a\,n\,n\,e\,l\,i\,n\,t\,h\,e\,g\,r\,o\,u\,p}{T\,o\,t\,a\,l\,n\,u\,m\,b\,e\,r\,o\,f\,c\,h\,a\,n\,n\,e\,l\,s\,i\,n\,t\,h\,e\,g\,r\,o\,u\,p}$$

### Seizure-Like Event Network Measures

Maximum distance of spread of SLE: The Euclidean distance from the electrode at which the initiation of SLE is observed in the brain slice to the furthest point from the initiation point. The row and column number are used as the x and y coordinates, respectively. The Euclidean distance between the x, y co-ordinates have no unit. It is multiplied by the electrode spacing in micrometers to determine the distance of spread of seizure-like activity in the brain slice.


M⁢a⁢x⁢i⁢m⁢u⁢m⁢d⁢i⁢s⁢t⁢a⁢n⁢c⁢e⁢o⁢f⁢s⁢p⁢r⁢e⁢a⁢d⁢(μ⁢m)

$$=\left[\sqrt{{\left(x_{2}-x_{1}\right)}^{2}+{\left(y_{2}-y_{1}\right)}^{2}}\right]\,X\,\left(E\,l\,e\,c\,t\,r\,o\,d\,e\,s\,p\,a\,c\,i\,n\,g\right)$$

Duration of SLE: This is calculated for each channel in a selected group. The difference between the end time and the start time of the seizure-like event in the selected time window of the “Channel Raster (Groups)” gives the seizure duration for that channel. The mean and maximum duration are calculated for each group from the duration of seizure-like activity of all channels in that group.


D⁢u⁢r⁢a⁢t⁢i⁢o⁢n⁢(s)=E⁢n⁢d⁢t⁢i⁢m⁢e⁢o⁢f⁢s⁢e⁢i⁢z⁢u⁢r⁢e⁢e⁢n⁢v⁢e⁢l⁢o⁢p-S⁢t⁢a⁢r⁢t⁢t⁢i⁢m⁢e

⁢o⁢f⁢s⁢e⁢i⁢z⁢u⁢r⁢e⁢e⁢n⁢v⁢e⁢l⁢o⁢p

Seizure propagation speed: For the selected time interval in the “Channel Raster (Groups),” the start time and end time of SLE are calculated for all channels in the group. The maximum distance of spread of the SLE is also calculated for that group. The seizure rate is the maximum distance of spread of the SLE divided by the mean difference in the start times of the seizure for each individual seizure.


S⁢e⁢i⁢z⁢u⁢r⁢e⁢v⁢e⁢l⁢o⁢c⁢i⁢t⁢y⁢(μ⁢m/s)

$$=\frac{M\,a\,x\,i\,m\,u\,m\,d\,i\,s\,t\,a\,n\,c\,e\,o\,f\,s\,p\,r\,e\,a\,d}{M\,e\,a\,n\,d\,i\,f\,f\,e\,r\,e\,n\,c\,e\,i\,n\,s\,t\,a\,r\,t\,t\,i\,m\,e\,o\,f\,a\,c\,t\,i\,v\,i\,t\,y\,i\,n\,t\,h\,e\,c\,h\,a\,n\,n\,e\,l\,s}$$

## Results

**FIGURE 1:**
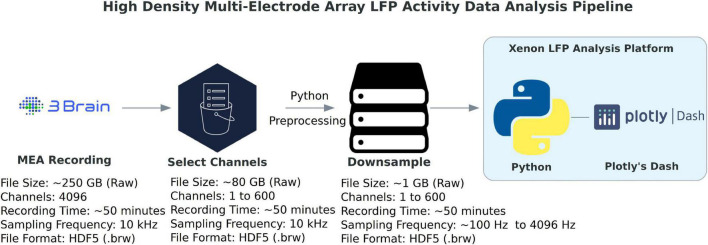
High-density multi-electrode array (HD-MEA) data-analysis pipeline. The data processing for LFP activity detection and network analysis starts by selecting a group of about 600 channels that overlay the brain slice or region of interest, which are exported from the original hdf5 measurement raw file (4,096 channels, sampled at 10 kHz) to a reduced hdf5 file. This reduced file is further downsampled from 10 kHz to a desired frequency. This will be the working hdf5 file for the Xenon LFP Analysis Platform. (3Brain Logo: ^©^Copyright 3Brain AG, Python Logo: ^©^Copyright Python Software Foundation, Plotly’s Dash Logo: ^©^Copyright Plotly).

**FIGURE 2:**
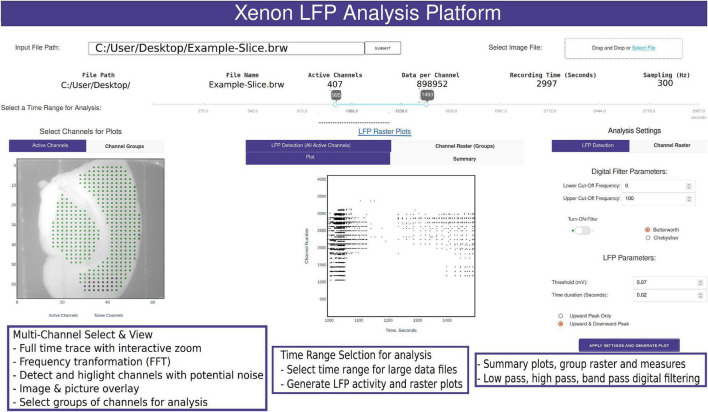
Snapshot of the analysis GUI features. A view of the analysis GUI which is rendered in an html browser built in Python using Plotly’s Dash. The GUI has several interactive features from individual and group channel selection, low-pass, high-pass, and band-pass filtering, viewing entire trace or a small section of the trace, Fast Fourier Transformation (FFT) of sections from selected traces, customized raster plots, small groups of channels, and generation of group summary measures.

The data processing pipeline for LFP activity and seizure analysis consists of three steps starting from the measurement file as shown in Figure 1. A typical measurement file consists of 4,096 channels recorded for about 50 min at a sampling frequency of 10 kHz. In the hdf5 format, the file size is about 250 GB uncompressed. As a first step, channels that overlay the brain slice are selected based on the desired resolution and exported using the 3Brain proprietary BrainWave4 software. This exported file consists of about 300 to 600 channels with the original sampling frequency and a reduced file size of 80 GB. The file size and number of channels selected in this step can vary depending on the recording sampling frequency, resolution required for the analysis, and recording time. Second, the extracted channels that overlay the brain slice from the previous step are downsampled in Python, this downsampled file maintains the same data structure and hdf5 format as the original recording, thus has backward compatibility with BrainWave4 software. The downsampled file is now ready for use with our custom interactive MEA Viewer—Xenon LFP Analysis Platform. The GUI is built in Python using the Plotly’s Dash library, which renders visualizations in a user-friendly web interface. A snapshot of the opening page of the web interface is shown in Figure 2. The analysis platform has the following key functions: (1) MEA Viewer Functions: This includes options to select and view individual channels, generate raster plots for all the channels, apply digital signal processing tools including FFT, low-pass, high-pass, and band-pass filters. (2) Channels Group Functions: This function has options to select three different regions or groups of channels, apply peak detection, generate custom raster plots, apply digital signal processing tools, and generate summary measures (SM) including LFP peak count per second, number of active channels, mean LFP peak amplitude, and mean LFP peak duration. (3) Seizure Detection and Analysis Functions: This function is an unsupervised automatic SLE detection on selected channel groups and analysis of metrics on seizures observed in the brain slice. Moreover, Python and Plotly’s Dash, which is based on object-oriented programming and reactive callbacks, provide options to customize or change the layout of visualization and data processing algorithms in the GUI, as per the user requirement within each of these functions. The GUI application is either hosted and run on a server or run in the local machine. While running the Python script in the local machine, by default the application can be accessed using a local host:8050 on a standard web browser.

### MEA Viewer Functions

A common challenge among researchers using large MEA recording platforms is that it is not easy to explore the raw data. The Xenon LFP Analysis Platform functions are aimed to facilitate exploring the raw data, including viewing entire time-series traces, apply threshold detection, and signal processing tools to individual and groups of channels. The entire platform is built for interactive explorations and analysis, while rendering the visualizations quickly in a few seconds. The time range selection (in Figure 2) is used to load and perform analysis on desired sections of the trace or the entire recording. Selecting channels for analysis is as easy as using the mouse to click on the green dots that are shown with the brain slice image in the background (Figure 2). Each point corresponds to the channel location, x and y axis referenced to the original row and column number on the MEA array. Multiple channels can be selected by holding down the shift key while clicking using the mouse. The selected channels automatically load and display for the given time range. Any changes in channel selection, time range selection, or analysis settings dynamically change the analysis measures and output displays. The analysis setting can be used to apply digital low-pass, high-pass and band-pass filters, modify default threshold and duration for peak detection and raster plot generation. All plots are interactive; they can be zoomed in, zoomed out, and downloaded as *. png files. Zooming small sections of the time-series in the LFP activity view will automatically generate FFT traces in an adjacent window. These functions are demonstrated in the Supplementary Video 1.

**FIGURE 3:**
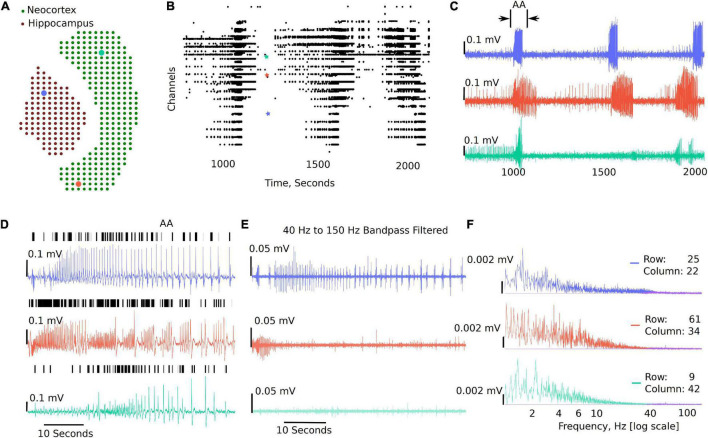
Example visualizations generated from the GUI including raster plots, time-series traces, LFP activity peaks, and time frequency transformations. **(A)** 407 channels selected for this analysis representing the MEA sensor spatial array, covering both the neocortex and the hippocampal regions. **(B)** Raster plot for all the channels in the working file irrespective of brain regions for a selected time range, demonstrating time points of when the activity occurs in the slice. **(C)** Three selected traces: the blue trace from the hippocampal region, the red trace from one end of the neocortex, and the aqua trace from the other end of the neocortex. **(D)** Zoomed in view of the first seizure [the region bracketed in panel **(C)** as AA], with the black dashes showing a peak find function within the GUI. **(E)** This demonstrates the ability to plot filtered traces along with the raw traces. **(F)** The amplitude frequency transformations for the traces in panels **(D,E)** (band-pass filtered). The filtered FFT spectrum for each is shown in purple.

Figure 3 shows a sample analysis demonstrating the MEA viewer functions in detail. In this example, 407 channels are exported from the original recording for analysis. The analysis file was downsampled from 10,000 Hz to 300 Hz. The green dots overlay the neocortex, and the electrodes corresponding to the brown dots overlay the hippocampus (Figure 3A). The raster plot in Figure 3B highlights LFP activity in the entire recording from LFP peak counts, which are user defined LFP parameters within the GUI for both duration and amplitude of the peaks (see Figure 2 and Supplementary Video 1). The raster plot is greatly affected by the different thresholds selected and the signal to noise ratio of the recordings; therefore, accuracy should be verified by the user as demonstrated in Figure 3D (see also Supplementary Video 1). The channels are arranged according to their x, y position in the row and columns from 1 to 4,096. The default threshold and duration for LFP activity is 0.07 mV and 0.02 s; however, the raster can be regenerated for a range of input values by modifying the parameters in the analysis settings (see Figure 2 and Supplementary Video 1), including generating raster after application of low-pass, band-pass, and high-pass filters. Figure 3C shows time-series traces from three electrodes (highlighted in Figure 3A); one from the hippocampus displayed in blue and one from either end of the neocortex displayed in red and aqua, respectively. It is interesting to note the difference in the activity pattern in the three traces at the same instant of time. While Figure 3C show traces for duration of the recording from the selected electrodes, a section of these traces can be selected to view on a faster timescale (Figures 3C,D AA), as shown in Figure 3D. The black vertical markers at the top of each trace shows LFP activity detected based on the given threshold and duration. This further highlights the difference in the activity pattern in the different regions of the brain slice at the same time. We can apply digital filters to the traces; for example a 40–150 Hz band-pass filter to view low and high-gamma activity (Figure 3E). We see the blue and red trace have some gamma components; however, the aqua trace does not have significant gamma components in the LFP activity. The time traces are interactive. To view spectrum plots (FFT), a small selection of the trace can be selected which automatically generates the FFT traces adjacent to the time-series traces (as shown in Figures 3E,F). The filtered and original traces are usually overlaid; however, to view one or the other, clicking on the legend selects/deselects the trace to view one or both at a time. When digital filters are applied, the amplitude spectrum of the band-pass-filtered and unfiltered (purple) traces are overlayed to show the effect of filtering (Figure 3F, unfiltered: purple, filtered: electrode-specific colors).

### Channel Group Functions

**FIGURE 4:**
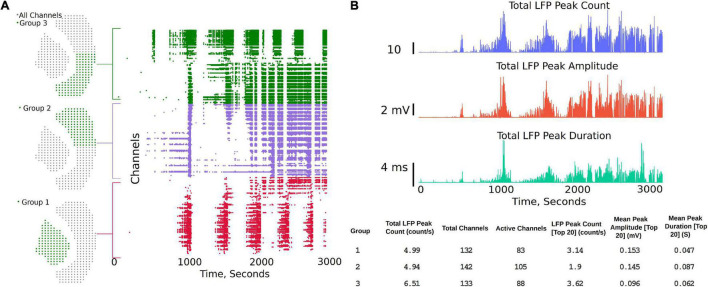
Channel groups and raster plot can be generated to visualize LFP activity in different regions of the brain slice. **(A)** Sensor locations corresponding to three different regions selected for analysis and the region-specific raster plots. Group 1 being the hippocampus, while groups 2 and 3 each being one half of the Neocortex. **(B)** Summary plots and measures that can be generated within the analysis platform.

The channel group functions are aimed at comparing two or three different regions of the brain slice and to compare LFP activity summary measures, while also generating a raster plot to study the activity pattern in different regions. The analysis starts with the “Channels Groups” tab (see Figure 2 and Supplementary Video 2). Channel groups can be selected by clicking on channels or by using the box or draw tool to select multiple channels at the same time. The groups tab enables selecting channels under three groups (Group1, Group 2, and Group3). The channels for each group are selected under their respective tab. Once respective groups and channels are selected, analysis settings can be modified from the default settings by clicking on “Apply Settings and Generate Plots,” which generates the raster plots and summary measures (SM) (Figures 4A,B). Some of the measures automatically calculated are also shown in Figure 4B, which include Total LFP Peak count/s and total channels within the group. Channels that have more than 20 LFP activity peaks count in the selected time interval are considered as active channels, and the last three measures are the LFP peak count/s, mean peak amplitude, and mean peak duration for the top 20 most active channels in each group. The channels considered the 20 most active channels are the 20 channels that have the most LFP peak counts for each selected group. As shown in the summary measures (SM) table, for Group 1 in Figure 4B (bottom), which includes 132 channels in the hippocampus, 83 channels are active, of which only the top 20 are used to compare the mean amplitude and mean duration in this example. Output of the top 20 channels is a default setting in the analysis platform which can be modified if required to include more channels or all the channels in the group. Further, the total activity, LFP peak amplitude, and peak duration are shown in the summary plot and includes all the channels in the group. The plots and summary measures (SM) can easily be regenerated for suitable selection of the time intervals by modifying the time range selection and channels in each group. The raster plot and LFP summary measures (SM) can also be generated following application of a digital filter. This can be useful for users interested in particular frequency bands. The front-end table displays a consolidated summary for channels in the group; however, the metrics for each individual channel in the group can be generated as a *. csv file for further analysis by clicking on the “LFP Raster Plots” hyperlink (see Supplementary Video 2). Note that this *. csv file will save in the default downloads location set by your browser, which is often the downloads folder for most PCs, but this can be changed by the user within the browsers. The saved analysis file for each channel consists of LFP peak count, mean peak amplitude (mV), peak duration (s), raw frequency power V^2^/Hz (for delta, theta, alpha, beta, and gamma), channel number, and group number for each individual channel for the time interval selected by the user. Each time the “LFP Raster Plots” hyperlink is clicked, a new *. csv is generated with the current analysis settings. The code can also be modified to save all log files continuously in a results folder if desired.

### Seizure Detection and Analysis Functions

#### Seizure Detection

**FIGURE 5:**
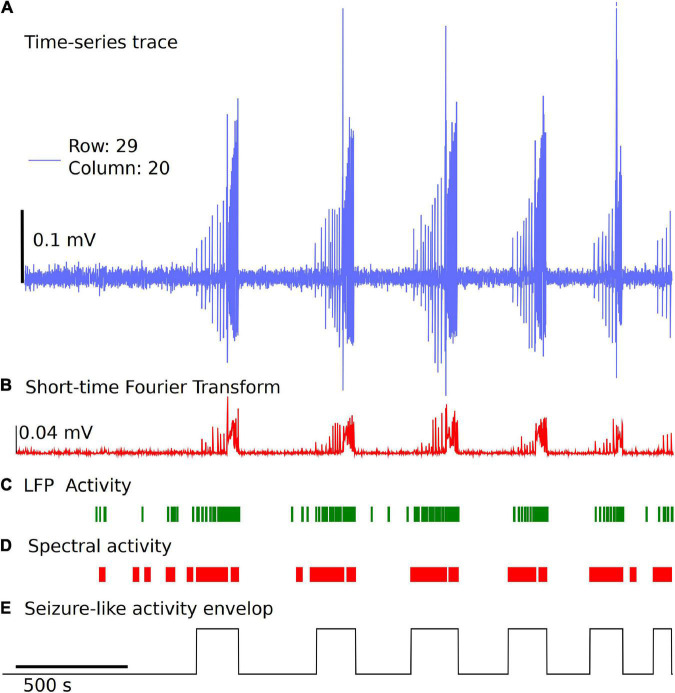
Simple and fast unsupervised seizure detection method. **(A)** The raw trace from the recording downsampled to 300 Hz frequency for a sample channel. **(B)** Spectral activity calculated from the Short-time Fourier Transform using Hanning Window, for a time window of 1 s with no overlap. **(C)** LFP activity is detected using a threshold of 6 standard deviations from the baseline voltage for each individual channel and a fixed duration of 0.035 s, followed by applying two sets of sliding windows (length 30 datapoints and 500 datapoints) to detect time regions of continuous activity. **(D)** Spectral activity is detected when the magnitude is greater than mean + 6 standard deviations from the baseline spectrum magnitude. The spectral activity is also passed through two sliding windows to detect regions of continuous spectral activity. **(E)** The overlapping regions of LFP activity and spectral activity of 10 s or more are used to identify the seizure envelop. The start of seizure is primarily identified using the time point when the spectral activity is greater than 6 standard deviations from the baseline.

Detection and classification of interictal, ictal or SLE can be quite challenging due to different types of epileptiform activity, variability from type of measurement paradigm (4-aminopyridine, low Mg^2+^, low Ca^2+^, high K^+^), and inherent experiment-to-experiment variability (Campos et al., 2018; Ghiasvand et al., 2020). In the Xenon LFP Analysis Platform, we introduce a simple method to detect SLE using changes in spectral activity and LFP activity in the traces. We found this method quick and easy to apply to many channels (>400 channels) at a time and compare the effects of different treatments. Moreover, this is efficiently implemented using numpy, scipy, and signal libraries in Python. The steps involved are illustrated in Figure 5.

We start with the time-series trace downsampled to 300 Hz. A reference section of 60 s is selected automatically in the first 5 min of the recording with no LFP activity, seizure-like activity, or electrical noise spikes. This is used as a baseline for spectral activity and voltage noise floor. The spectral magnitude is calculated using the Short-time Fourier Transform (STFT), with a few variable parameters that can be set or standardized in the analysis platform, including length of time segment, window, and overlap points (Figure 5B). The default settings for STFT in the analysis use a time segment length of 1 s, and a Hanning window with no overlap. For the spectral component magnitudes as a function of time, within each time window (1 s), the sum of all frequency components less than half the sampling frequency is used for event detection. Two sliding windows of dimension 30 datapoints and 500 datapoints are applied to the spectral activity peaks and LFP activity peaks independently to detect regions of continuous seizure-like activity and time regions of no activity (Figures 5C,D). This has a few parameters that can be standardized based on the experiment paradigm. In the examples discussed, we use mean + 6 standard deviations from the baseline spectral magnitude to detect high spectral activity and 6 standard deviations from the baseline voltage as the threshold and a fixed duration of 0.035 s for LFP peak activity detection. The sliding window length (30 × 1 and 500 × 1) and cutoff values for automatically detecting spectrally active time regions post windowing can also be standardized. Once we have the time points of continuous spectral activity and LFP activity, we use overlapping points of both spectral activity and LFP activity to detect the seizure envelop. In general, the start of SLE is primarily detected when the spectral activity exceeds six standard deviations from the baseline and has continuous spectral and LFP activity for a minimum of 10 s. This again can be modified based on the experiment paradigm. If some seizures are closely spaced, parameters can be changed to a different value based on user preference. Once we have the seizure envelop with start and end times, we use this to further calculate the rate of seizure spread, distance of spread of seizure within a region of a brain slice using the group selection as discussed in the next section. This being an unsupervised method, and the variability of the nature of seizure-like activity in different regions of the tissue and between experiments, this may require manual verification by selecting a few channels and checking if the automatic envelop detect has good accuracy. We noticed that when the signal to noise ratio is high, and when clear LFP activity and spectral activity is detected, the algorithm performs well, but may need some adjustments to the parameters when the signal to noise ratio is low or LFP activity is not clearly differentiable.

#### Seizure Analysis

**FIGURE 6:**
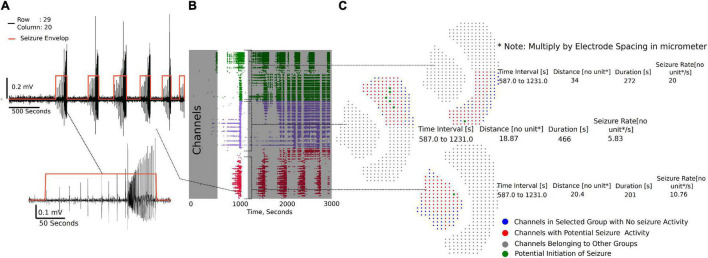
Seizure activity tracking over space and time. **(A)** Seizures in individual channels in a group are automatically detected. Their respective start and end times can be tracked across channels in that group. **(B)** Regions of the raster between time intervals can be selected as demonstrated to generate seizure maps of selected brain regions within the interval. **(C)** Seizure map for the time interval selected and channels in the group, including initiation site of the seizure, maximum distance the seizure spread from the initiation point, duration of the seizure, and the rate of seizure spread across the tissue.

The channel group raster is required to perform the seizure detection and analysis. Each group has a separate tab (Supplementary Video 3) under which individual channels can be selected to view seizure-like activity highlighted by the envelop (Figure 6A). Figure 6B demonstrates the raster plot for three different groups. Using the raster, a region can be selected with a potential SLE, as shown in Figure 6B (non-gray section), to generate summary measures and a visual of the channels that have an SLE within the selected section (Figure 6C). The channel dots highlighted in red are channels in the respective group that have an SLE, the blue dots are channels that did not participate in the SLE, while the gray dots have not been selected. The time interval shown in the summary table in Figure 6C is the selected time interval in the raster plot (Figure 6B non-gray section). The distance, duration, and seizure rate are calculated from the start and end times of seizure envelop in each of the channels in the group for the selected (zoomed in) seizure.

Three metrics are calculated from the seizure envelop for all channels in the group: distance of spread, duration, and seizure propagation speed. The spatiotemporal origin of the seizure within a group is identified as the channel that first had spectral activity above the set threshold. This timestamp and the location of the channel is used to further calculate the distance and rate of the seizure spread. For example, in Figure 6C, it is the maximum distance from the green dot to the furthest red dots. If more than one channel is highlighted green, then they have similar start times, and the maximum distance from each point is calculated to find the overall maximum distance. The blue dots do not have a seizure-like event and are not included in the calculation. The x, y position on a 64X64 grid places the channels at 1 unit dimension from each other. The array spacing in micrometer is multiplied by the distance and seizure rate to get the final measure in micrometer and micrometer/second, respectively.

**FIGURE 7:**
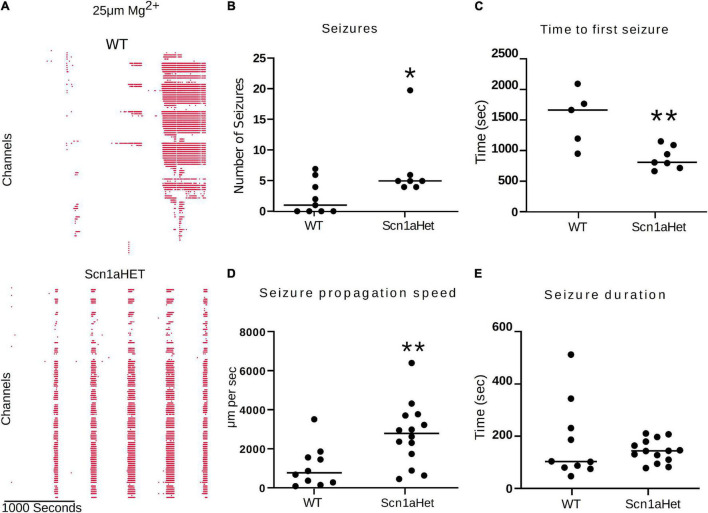
Scn1aHet mice have an altered seizure pattern in the low Mg^2+^ model. **(A)** Example raster plots from a control brain slice and a brain slice from Scn1aHet mice. **(B)** Scn1aHet mice have significantly more SLE than littermate controls (Mann–Whitney test, *p* = 0.04, *n* = 7–9 slices). **(C)** From the brain slices that displayed SLE, Scn1aHet demonstrated a significant increase in time to first seizure compared to littermate controls (unpaired *t*-test, *p* = 0.006, *n* = 5–7 slices). **(D)** SLE from the Scn1aHet propagate significantly faster than seizures in the littermate controls (Mann–Whitney test, *p* = 0.0059, *n* = 10–14 seizures from 5 to 7 slices). The first two seizure from the slices that had SLE were used in this analysis. **(E)** Seizure duration was not different between Scn1aHet and littermate controls (Mann–Whitney test, *p* = 0.66, *n* = 10–14 seizures from 5 to 7 slices). The first two seizures from the slices that had SLE were used in this analysis. *P < 0.05, **P < 0.01.

We next used this seizure tracking function to examine if neocortical seizure-like events from brain slices from Scn1aHet mice, which are heterozygous for NaV1.1, have an altered phenotype in the low Mg^2+^ model of acute ictogenesis. The example raster plots demonstrate a likely difference in number of seizure-like events between WT littermates and the Scn1aHet animals (Figure 7A). Further analysis revealed that the Scn1aHet mice do have significantly more seizures than the WT littermates over the course of the 50-min recording (Figure 7B). Furthermore, we found that the start time to the first seizure-like event was significantly sooner in the Scn1aHet animals compared to controls; further demonstrating an increased seizure phenotype in animals with a deficit in NaV1.1 expression (Figure 7C). Using our novel tracking algorithm for seizures within our GUI, we compared the speed of seizure propagation in brain slices from control mice versus the Scn1aHet mice. Interestingly, this analysis demonstrated a significantly faster rate of seizure propagation in brain slices from the Scn1aHet mice compared to control (Figure 7D). There was no significant difference found in the duration of the seizures between the control and Scn1aHet mice (Figure 7E). This data demonstrates novel phenotypic features of the Scn1aHet mice; a decreased time to the appearance of the first seizure-like event and an increased rate of seizure spread through the tissue, likely due to deficits in feed-forward inhibition provided by the somatostatin and parvalbumin interneurons (Trevelyan et al., 2007; Cammarota et al., 2013; Parrish et al., 2019). These new analysis features provided by the Xenon LFP Analysis Platform provide new and exciting ways to understand phenotypic differences in transgenic animals, understand how pharmacology impacts neuronal network activity over space and time, and is customizable to fit any researcher’s needs.

## Discussion

The Xenon LFP Analysis Platform aims to produce an interactive application with high-quality visualization rendered on a web browser, using open-source libraries (Python and Plotly’s Dash) that can be standardized to an individual’s research requirements. In the examples shown in the results section, we provide a snapshot of simple visualization and signal processing tools; however, this can be expanded and customized to include additional features as per the users’ requirements by building simple data analysis models/functions and rendering them using callbacks in Plotly’s Dash. The data models with Xenon LFP Analysis Platform enable creating summary measures for comparisons and visualizations on the browser, creating an interactive toolbox for viewing millions of datapoints at a time, to extract meaningful results and conclusions from the measurements. Furthermore, the application is scalable to larger datasets with the ability to build functions that selectively read from small chunks of data from the hdf5 array rather than loading the entire dataset into memory for rendering on the browser. However, it should be noted that one of the drawbacks of hdf5 files to store HD-MEA data is that using single dimension large arrays to store data makes indexing and selectively reading channels very inefficient and difficult to parallelize (Rossant, 2016a,b; Dragly et al., 2018). Most HD-MEA measurement systems use the hdf5 files system to record/write data to disk, which provides advantages for portability of data but limits data analysis pipelines to parallelize signal processing tasks on distributed systems or multicore processors and GPUs. Some cases require reading the entire array to memory for extracting a group of channels to apply a band-pass filter or Fast Fourier Transforms (FFT). The current working file size on the analysis platform is limited by local system RAM. Future work can extend the current platform to include a data pipeline to work with larger files of 250 GB or more exceeding the system memory, using parallel computing algorithms for signal processing and visualization tasks including filtering, FFT analysis, and spike sorting on a larger scale, which is a developing research area for computational neuroscience that requires more exploration (Jonathan and Sahani, 2019; Street, 2021).

The spatiotemporal resolution of HD-MEA recordings on brain slices provides high-quality data, while also presenting big data challenges in visualization and analysis, including extracting meaningful reproduceable results. This can further be complicated when testing long-duration drug protocols to include multiple compounds at different concentrations resulting in terabytes of data that can become overwhelming to analyze and compare (Perkel, 2018). There is always a need for simple data pipelines and new analysis platforms that are open source, user friendly, scalable, and portable that can produce repeatable analysis results for ease of comparison between paradigms and datasets (Mouček et al., 2014; Sejnowski et al., 2014). Standardization of analysis tools to compare different drug protocols is key to make sense of terabytes of data collected using different compounds, concentrations, and drug-treatment effects (Sobolev et al., 2014). The Xenon LFP Analysis Platform enables this by setting up standard functions with customizable parameters to generate raster plots and LFP metrics. This includes unsupervised methods to detect seizure-like activity. There are three key groups of measures: (1) summary measures relating to all channels in each recording, (2) metrics relating to channel groups, and (3) seizure-like event measures tracked for selected regions in the raster plots for channel groups. In the first step, LFP activity raster and activity count for all active channels are summarized in the “LFP Detection (All Active Channels)” tab (Figure 2). This data is not saved and is just rendered on the browser for viewing, which may be useful to quickly review the recording. This is also linked to the selected time range and analysis settings, including threshold, duration, and digital filter parameters. In the second step, channel groups or select regions of the brain slice may be selected along with a specific time range to generate custom raster plots, along with metrics like the number of active channels, LFP peak count, mean LFP peak amplitude, mean LFP peak duration [“Channel Raster (Groups)”]. As shown in the results section, this is particularly useful to compare different regions of the brain slice or different time regions within a recording to explore the effects of a drug application. In addition to viewing, all measures for individual channels in each group can be saved as a *. csv file for further analysis. In the third step, the raster generated in step two can be used to select specific time points of activity to view and analyze LFP and SLE activity (Figure 5). These measures track network activity based on seizure envelops, start times, and end times. This being an unsupervised method, and due to the variability in measurement for different brain slices and protocols, user intervention may be required in some cases to check the activity envelop and careful selection of activity regions in the raster plot. It is our hope that making this analysis platform fully open source will allow others to add functions that enhance its utility for all and aid in addressing some limitations of this current GUI, such as aspects of the analysis requiring some user intervention and finding alternative approaches to streamline analysis of even larger data sets. One limitation of the Xenon LFP Analysis Platform may be found in our seizure detection methodology due to the frequency spectrum available to us from the 2D C-MOS electrodes that does not penetrate the brain slice, but rather the brain slice is laid onto the electrodes. This current technique does not generally allow for collection of higher frequency components that can be recorded from acute brain slices with penetrating electrodes, where high frequency components, such as gamma, can be used for seizure detection (Weiss et al., 2015). As detailed in Figure 5, our seizure detection is based on LFP activity and spectral activity, using the frequency components that can be found within our recordings, where the most dominant are between 1 to 30 Hz. Nevertheless, we have found our method to be reliable, time efficient, and robust at detection of seizures for 100 to 1,000 s of channels at once. However, our seizure detection method cannot discriminate completely between the pre-ictal period and the ictal period, meaning that pre-ictal discharges may be included within the seizure envelop. To truly distinguish between the pre-ictal and ictal period within a recording, one must use frequency components well above 100 Hz (Schevon et al., 2012).

With the advent of larger recording systems, allowing for up to six brain slices and over 1,000 channels per slice during a single recording session, tools like this GUI are timely. These new systems will allow for immense screening of transgenic animals to elucidate aberrant network behavior (Mackenzie-Gray Scott et al., 2022) and large-scale drug screening of biological tissue. Furthermore, with epilepsy and other disorders, there is a need to understand how different brain regions interact with each other when challenged in media that induces increased network activity or when stimulated electrically or optogenetically (Rafiq et al., 2003; Codadu et al., 2019b; Cela and Sjostrom, 2020). While we now have the recording platforms to facilitate these research needs, we are still limited by analysis tools. Here we directly address some of these needs in our GUI and set important groundwork for further developments within this platform. We also perceive that this GUI will be useful in other large-scale electrophysiological recording systems where the researcher wants to understand interactions between LFP activity at different recording sites over space and time. For example, it would be particularly interesting to visualize multichannel human EEG recordings within the framework of this GUI, which could provide easy and efficient visualization of channel recruitment during various behavioral states with the current built-in features and custom additions.

Overall, the Xenon LFP Analysis Platform introduces a standard approach to analyze large HD-MEA recordings, using high-quality visualization rendered on a browser, simple algorithms, and metrics, with many customizable features and options for researchers. We demonstrate the utility of this new analysis platform with *ex vivo* data and demonstrate a novel finding in a low Mg^2+^ model of epilepsy from Scn1aHet animals. Brain slices from the Scn1aHet animals display an increased rate of seizure propagation compared to slices from WT littermates. Using hundreds of channels to map spreading activity, such as seizures, adds another important tool in the hands of neuroscientists and will complement low-resolution traditional imaging techniques, such as Ca^2+^ imaging and dye-based voltage imaging. We hope this GUI will serve as a tool for collaborative work between research labs to contribute add-ons and share results and findings.

## Data Availability Statement

The original contributions presented in this study are included in the article/Supplementary Material, further inquiries can be directed to the corresponding author/s.

## Ethics Statement

This animal study was reviewed and approved by Xenon Animal Care Committee; Xenon Pharmaceuticals.

## Author Contributions

RP conceived the work. AM, NC, and RP designed the computational methods and edited and approved the final draft. AM wrote the code and designed the visualizations. RP collected the data. AM and RP analyzed the data and wrote the manuscript. All authors contributed to the article and approved the submitted version.

## Conflict of Interest

The authors declare that this study received funding from Xenon Pharmaceuticals Inc. The funder was not involved in the study design, collection, analysis, interpretation of data, the writing of this article or the decision to submit it for publication.

## Publisher’s Note

All claims expressed in this article are solely those of the authors and do not necessarily represent those of their affiliated organizations, or those of the publisher, the editors and the reviewers. Any product that may be evaluated in this article, or claim that may be made by its manufacturer, is not guaranteed or endorsed by the publisher.

## Footnotes

1
https://github.com/MicroBrew09/xenon-lfp-analysis

## Supplementary Material

The Supplementary Material for this article can be found online at:

Supplementary Video 1 MEA Viewer Functions (Xenon LFP Analysis).

Supplementary Video 2 MEA Group Functions.

Supplementary Video 3 Seizure-like Activity (Xenon LFP Analysis).
